# Supplementary material for: Active site specificity profiling datasets of matrix metalloproteinases (MMPs) 1, 2, 3, 7, 8, 9, 12, 13 and 14
Source: Data Brief. 2016 Feb 22;7:299–310. doi: 10.1016/j.dib.2016.02.036 (PMC4777984; doi:10.1016/j.dib.2016.02.036)
Supplement: Supplementary file 10 — Supplementary material [file mmc10.zip › WebPICS_hMMP3_G_1%/P1prime.html]

 

PICS results


|  |  |
| --- | --- |
| **P1prime\_C**  10 in 151 sites   6.6 %    effects > 10 perc. pnts.  (vice-versa in brackets)  P3\_A: 26.8 (13.4)   P2\_L: 28.7 (16.9)   P1\_D: 20.1 (13.4)   P2prime\_I: 18.1 (10.1)   P3prime\_L: 28.7 (16.9) |  |
| **P1prime\_I**  26 in 151 sites   17.2 %    effects > 10 perc. pnts.  (vice-versa in brackets)  P2\_A: -11.5 (-10.3)   P2\_C: 10.8 (39.9)   P1\_N: -12.1 (-13.0)   P2prime\_H: 12.1 (62.8)   P2prime\_L: 14.3 (19.6)   P3prime\_A: 13.6 (13.6)   P3prime\_G: 11.9 (28.3) |  |
| **P1prime\_Q**  10 in 151 sites   6.6 %    effects > 10 perc. pnts.  (vice-versa in brackets)  P2\_F: 16.0 (26.7)   P1\_A: 20.1 (13.4)   P3prime\_G: 12.7 (11.6)   P3prime\_I: 12.1 (10.1) |  |
| **P1prime\_V**  21 in 151 sites   13.9 %    effects > 10 perc. pnts.  (vice-versa in brackets)  P3\_A: 10.6 (11.1)   P3\_I: 15.2 (24.6)   P2\_F: 15.0 (52.8)   P1\_C: 12.3 (86.1)   P1\_Q: 10.4 (16.9)   P2prime\_I: 16.7 (19.4)   P2prime\_T: 12.4 (26.1) |  |
| **P1prime\_Y**  5 in 151 sites   3.3 %    effects > 10 perc. pnts.  (vice-versa in brackets)  P2\_H: 16.0 (13.4)   P1\_H: 16.7 (16.7) |  |
